# Catalytic Activity of 2-Imino-1,10-phenthrolyl Fe/Co Complexes via Linear Machine Learning

**DOI:** 10.3390/molecules29102313

**Published:** 2024-05-15

**Authors:** Zubair Sadiq, Wenhong Yang, Md Mostakim Meraz, Weisheng Yang, Wen-Hua Sun

**Affiliations:** 1 Key Laboratory of Engineering Plastics, Beijing National Laboratory for Molecular Science, Institute of Chemistry, Chinese Academy of Sciences, Beijing 100190, China; zubairzr@iccas.ac.cn (Z.S.); meraz@iccas.ac.cn (M.M.M.); 2 University of Chinese Academy of Sciences, Beijing 100049, China; 3 PetroChina Petrochemical Research Institute, Beijing, 102206, China

**Keywords:** catalytic activity, descriptor, ethylene oligomerization, Fe/Co complexes, machine learning

## Abstract

In anticipation of the correlations between catalyst structures and their properties, the catalytic activities of 2-imino-1,10-phenanthrolyl iron and cobalt metal complexes are quantitatively investigated via linear machine learning (ML) algorithms. Comparatively, the Ridge Regression *(RR)* model has captured more robust predictive performance compared with other linear algorithms, with a correlation coefficient value of *R*^2^ *=* 0.952 and a cross-validation value of *Q*^2^ *=* 0.871. It shows that different algorithms select distinct types of descriptors, depending on the importance of descriptors. Through the interpretation of the *RR* model, the catalytic activity is potentially related to the steric effect of substituents and negative charged groups. This study refines descriptor selection for accurate modeling, providing insights into the variation principle of catalytic activity.

## 1. Introduction

Tremendously growing interest in industrial and household applications of synthetic polymers such as polyolefins derived from monomers like alpha-olefins has made their demand worth noting, and opened new eras of scientific and industrial research. Though most commercially available polyolefins are produced by using heterogeneous Ziegler-Natta catalysts [1], currently homogeneous catalysts [2,3] are gaining attraction in the market. These well-defined catalysts, particularly cobalt and iron complexes, pioneered by Brookhart [4] and Gibson [5] in the late 1990s, offer remarkable performance in ethylene oligo-/polymerization, yielding linear polyethylenes or alpha-olefins [6].

Oligomerization of ethylene stands as a significant industrial method for generating linear-alpha olefins, which serve as key components in the production of detergent alcohols, lubricant additives, surfactants, plasticizers, and polymer modifiers [7]. Transition metal complexes are significantly involved in the process of oligo-/polymerization of ethylene, and when it comes to catalytic effectiveness, the activity of reaction holds a paramount position. As a result, substantial research has been dedicated to developing and creating novel, and high-performance catalysts for this special purpose.

**Fe** and **Co** complexes featuring N^N^N-type phenanthroline backbone ligands [8], derived from the bisiminopyridine structure [9,10], have exhibited superior catalytic performances in ethylene oligomerization. Scientific research on these late-transition metal complexes has given new insights into potential improvements in catalytic activity and polymer control. Notably, the successful use of the 2-imino-1,10-phenanthrolyl-iron catalyst in large-scale production (∼50,000-ton scale annually) of high-quality linear alpha-olefins (LAOs) [8,11], followed by an expansion to 200,000-ton processes in China (in construction since November 2021) highlights the promise of late transition metal technology and its revitalization in the industry. Despite these efforts and achievements, the potential principle underlying the variation in the catalytic activity is still needed at the molecular level.

Catalytic activity of transition metal complexes is primarily linked to the catalyst’s structure, encompassing both electronic and steric effects. The former is characterized by the ability of a substituent to either donate or withdraw electrons while the latter corresponds to factors like the size of the substituent, bond length and angle, and the atomic radius of the metal atom [12,13,14]. Earlier studies employing Multiple Linear Regression Analysis (*MLRA*) predicted the catalytic activities of late transition metal complexes well by taking into account structural descriptors from both steric and electronic aspects. Take, for instance, the fact that the catalytic efficiencies of four distinct groups of metal complexes featuring 2-azacyclyl-6-aryliminopyridyl ligands are explored by considering electronic (effective net charge, *Q*_eff_; Hammett constant, *F*) and steric effects (bite angle, *β*; open cone angle, *θ*) with primary focus on how different substituents on N-aryl groups influence the catalytic performance. The computed catalytic activities present a remarkable degree of consistency with the experimental data [15].

In another study, the variations in catalytic performances within two groups of metal complexes comprised of 2-imino-1,10-phenanthrolyl ligands are examined via *MLRA*. Notably, robust correlations are evident among complexes having the same metal atom but different ligand substitutions, whereas metal complexes with different central metal atoms (**Fe**, **Co** and **Ni**) showed comparatively weaker correlations [16]. In subsequent investigations, the changes in catalytic effectiveness were calculated between iron and cobalt complexes containing bis(pentamethylene)pyridyl ligands. Herein, the number of descriptors is increased to seven for improved and better predictive accuracy. The combination of two descriptors (*Q*_eff_ and *β*) exhibited a strong correlation with experimental activity values along with the correlation coefficient (*R*^2^) value over 0.934 [17].

Combined experimental and modeling studies are also conducted to probe the influence of substituents on the catalytic activities of symmetric 2,6-bis(imino)pyridyl cobalt complexes incorporating dibenzopyran [18], and α,α′-bis(imino)-2,3:5,6-bis(pentamethylene)pyridine-iron(II) chloride complexes [19]. The findings in the former series of complexes revealed that the influence of substituents on activity is mainly driven by steric effects, which implies that higher values of open cone angle are conducive to higher activity levels, whereas the electronic effect becomes a dominant factor in the later set of complexes.

As discussed above in previous reports on linear regression [15,16,17,18,19], *MLRA* alone has been used as a potent tool for quantitatively predicting the catalytic activities of late-transition metal complexes in ethylene oligo-/polymerization reactions. This study is inspired by the possibility of capturing more and robust correlations via other available linear algorithms such as Ridge Regression *(RR)* [20], Least Absolute Shrinkage and Selection Operator *(LASSO)* [21], and Elastic Net Regression *(EN)* [22]. So in this study, we build upon this foundation by introducing additional linear algorithms besides *MLRA,* aiming to find super predictive powers for a series of 2-imino-1,10-phenanthrolyl iron and cobalt complexes [23,24]. Herein, we also broaden the range of independent variables from descriptors of electronic and steric effects to descriptors calculated by resources such as Codessa (version 2.7.2) [25] and PaDEL (version 2.21) [26]. As anticipated, the investigation unveils that the *RR* regression model effectively captures improved correlation patterns. Moreover, different algorithms select varying types of descriptors, based on their relative importance. *RR* and *MLRA* selects all three types of descriptors, while *EN* and *LASSO* selects Codessa and self-defined type descriptors only, due to the different regularization strengths implemented across different algorithms.

## 2. Results and Discussions

### 2.1. Dataset

**Scheme 1:**
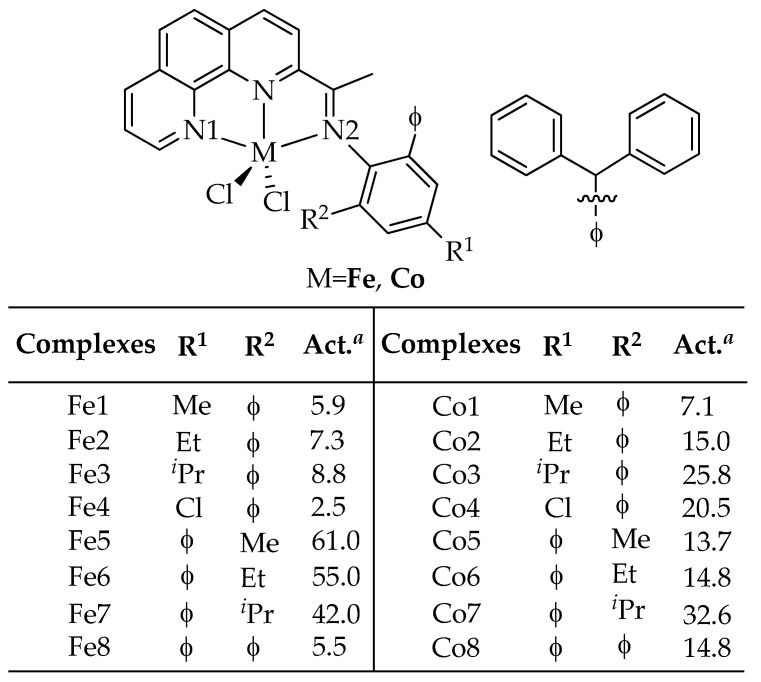
A dataset of benzhydryl-modified 2-imino-1,10-phenanthrolyl iron and cobalt complexes. *^a^* 10^4^ g·mol^−1^·h^−1^.

A dataset of 16 metal complexes consisting of benzhydryl-modified 2-imino-1,10-phenanthrolyl iron complexes (**Fe1**–**Fe8**) and cobalt complexes (**Co1**–**Co8**) was sourced from recent experimental reports [23,24], as shown in Scheme 1.

Firstly, the complexes are optimized via the Density Functional Theory (DFT) and the geometries are compared with experimental crystal structures as shown in Table S1 for **Fe** complexes and Tables S2–S4 for **Co** complexes, respectively. It can be seen in Table S1 that the values of the standard deviations of the bond lengths and bond angles for **Fe7** are lower at the quintet state than other spin states, indicating that the geometry at quintet is closer to the experimental structure. Similarly, as seen in Tables S2–S4, the geometries at the quartet state for **Co3**, **Co7**, and **Co8** show the lowest standard deviation values for bond lengths (1.06, 0.96, and 1.07) and bond angles (3.53, 9.15, and 3.98), and thus the quartet state is considered for optimization of **Co** complexes.

### 2.2. Calculation and Selection of Descriptors

The optimized geometries of iron and cobalt complexes, respectively, at quintet and quartet spin states are selected for calculating descriptors using three different sources, including self-defined type, Codessa, and PaDEL. The so-called self-defined type refers to descriptors developed in our previous studies as mentioned in the Introduction section. Therefore, there are seven self-defined structural descriptors calculated from electronic and steric effects, namely, effective net charge (*Q*_eff_), Hammett constant (*F*), HOMO-LUMO (Δ*ɛ*_1_, Δ*ɛ*_2_) energy gap, energy difference (Δ*E*), open cone angle (*θ*), and bite angle (*β*). The calculated results are obtained based on the optimized structures of each complex and listed in Table S5. To investigate the importance of each descriptor, the correlations between descriptors and catalytic activities are calculated as shown in Table S6. It could be seen that only 3 descriptors out of 7 (*β*, Δ*E*, *θ*) showed certain correlations with catalytic activity and hereby are considered to create a common pool of descriptors.

More than three-hundred descriptors are calculated by using Codessa for each complex. The descriptors are pre-screened and selected via the heuristic method (HM) [27] based on the two following criteria: each pair of descriptors is highly inter-correlated, with a threshold value of correlation coefficient (*R*^2^) > 0.99; the descriptor and target have a lower cross-correlation, with a threshold value of *R*^2^ < 0.01. Initially, 269 descriptors are removed after pre-screening. Then, the inter-correlation of the remaining 104 descriptors is calculated, which eliminated 42 descriptors, leaving behind 62 descriptors. To overcome the risk of overfitting, the number of descriptors should be less than half of the dataset. So, the correlations with catalytic activity by using different numbers of descriptors are calculated as the number of descriptors decreasing from 7 to 3, shown in Table S7. It is clear that by using 7 descriptors, the correlation is very good with *R*^2^ value of 0.991; then, 7 descriptors are chosen to add into the common pool of descriptors. Detailed information on the 7 descriptors via Codessa is given in Table S8.

For PaDEL descriptors, more than one-thousand descriptors are generated for each complex. Initially, descriptors containing missing or similar values are manually excluded, resulting in a remaining set of 790 descriptors. Then the remaining descriptors are further pre-screened via HM as well, leaving 382 descriptors. Further selection for descriptors is carried out by using the Partial Least Square-Variable Importance of Projection (PLS-VIP) method [28,29,30]. Based on the VIP value of 0.85, the number of descriptors is reduced gradually from 382 to 7 descriptors as shown in Table S9. Considering the prediction powers and errors, 7 descriptors are selected for adding into the common pool of descriptors. Detailed information on the 7 descriptors via PaDEL is given in Table S10.

Subsequently, a pool of 17 descriptors is built such that 7 descriptors are from Codessa, 7 descriptors are from PaDEL, and 3 descriptors are self-defined, as shown in Table 1. To clarify different types of descriptors, the sequence number of Codessa type is highlighted in red, and blue and green for PaDEL and self-defined types, respectively. To check the dependence among descriptors, the correlation for each pair of selected descriptors along with catalytic activity is presented in Figure S1**.** The low correlation values indicate their independent nature. To overcome overfitting, the number of descriptors is considered to range from 7 to 3 in order to select optimum results. A feature-selection technique, Recursive Feature Elimination (RFE), is used to select the desired descriptors via the different linear models. For more robust model predictions, the whole dataset is used. The shuffle split cross-validation method (n_splits = 4) is used to validate model performance.

### 2.3. Prediction via Four Linear ML Models

To build the model to predict the catalytic activities of **Fe**/**Co** complexes, four linear algorithms are considered: *MLRA*, *LASSO, EN*, and *RR*. Table 2 and Table 3 show the results for prediction and validation power using different numbers of descriptors by each model.

In Table 2, it can be seen that for the *MLRA* model, the catalytic activities can be well predicted by using 7 descriptors, and the correlation coefficient (*R*^2^) value is 0.973, then it starts to decrease until 0.831 when using 3 descriptors. The cross-validation values (*Q*^2^) decrease from 0.817 to 0.547 correspondingly. For the *LASSO* model, the correlation *R*^2^ values range from 0.949 to 0.917 as descriptors decrease from 7 to 3, and the corresponding *Q*^2^ values are from 0.755 to 0.743. Compared with the *MLRA* model, the *R*^2^ values are lower by 7 descriptors, but become higher by using a smaller number of descriptors, as from 6 to 3; meanwhile, the cross-validation values present around 0.75, indicating a more robust *LASSO* model.

In Table 3**,** it can be seen that the correlation coefficients for the *EN* model range from 0.994 to 0.942 and values of *Q*^2^ fall in the range of 0.944 to 0.774 as a function of the number of descriptors. The prediction and validation values are both higher than that of the *LASSO* model under each number of descriptors. As to the *RR* model, the correlation value is 0.996 by 7 descriptors, which is the highest compared with the other three models. Furthermore, the correlation remains the highest as the values of *R*^2^ change from 0.995 to 0.942 with the number of descriptors from 6 to 3. Meanwhile, the cross-validations present very high values falling in the range of 0.960 to 0.871 with the variation in descriptor numbers.

From the results of these linear models, it is clear that the prediction and validation performance increases in the following order: *MLRA* < *LASSO* < *EN* < *RR*. The *RR* model provided the best performance even for smaller numbers of descriptors, along with smaller prediction errors. The performance of the *MLRA* algorithm is seen to present the lowest performance in terms of explaining variances and generalizations. It is clear that when the number of descriptors is 3, the correlation is 0.831, which is lower than 0.9. The difference between the four algorithms lies in the penalty term in loss function. The better performance exhibited by the *RR* model is attributed to its ability to produce better generalizations and deal with multicollinearity by preventing overfitting.

**Figure 1:**
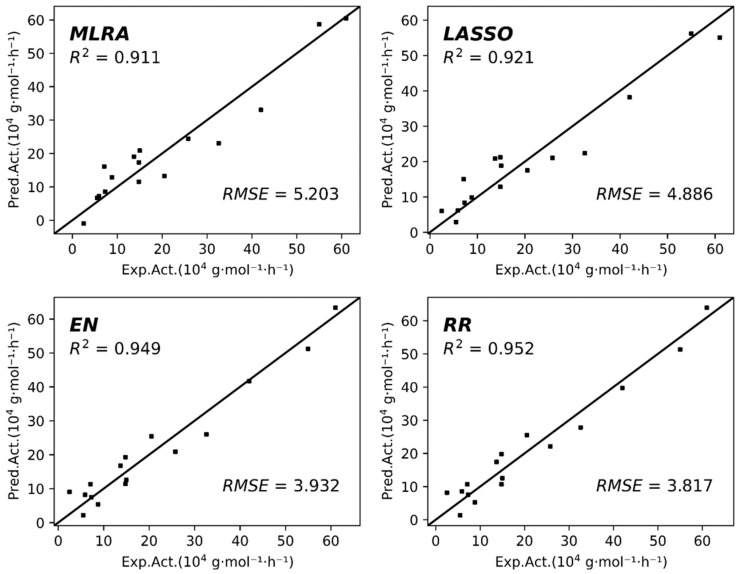
Comparison between predicted and experimental values of catalytic activities by different linear models using four descriptors.

As the number of descriptors ranges from 7 to 3, all the models present good *R*^2^ and *Q*^2^ values, except for the *MRLA* model under 3 descriptors. To simplify the model, a lower number of descriptors is considered given similar prediction and validation powers. Therefore, the number of 4 descriptors is selected to build the linear ML models. The predicted catalytic activities for all the algorithms are given in Table S11 by using 4 descriptors. Correspondingly, the comparison between the predicted catalytic activities and observed data in experiments is plotted in Figure 1.

Figure 1 illustrates the comparison between predicted and experimental catalytic activities across four ML models. A closer alignment of data points with the diagonal line signifies lower errors generated by the machine learning models. This close correspondence between predicted and actual values validates the model’s capacity to forecast catalytic activities effectively. Notably, Figure 1 also offers intriguing insights: in comparison to the other three models, the *RR* model has a relatively high concentration of data points near the diagonal, which are slightly further away in the case of the *EN* model and farther away in the *LASSO* and *MLRA* models. The *MLRA* and *LASSO* models show more scattered data points as compared to the diagonal, indicating larger bias amplitudes. Conversely, the *RR* model demonstrates relatively small bias amplitudes and a higher concentration of data points in proximity to the diagonal line as compared to the *EN* model.

### 2.4. Interpretation of the Models

In order to interpret the model, the contributions of descriptors are analyzed. The analysis shows that 12 descriptors (Nos. **1**, **3**, **4**, **5**, **6**, **8**, **9**, **11**, **12**, **15**, **16**, **17**) out of 17 are selected by different algorithms and 5 descriptors are never selected by any of the algorithms. Among the selected descriptors, there are 5 Codessa descriptors (Nos. **1**, **3**, **4**, **5**, **6**), 4 PaDEL descriptors (Nos. **8**, **9**, **11**, **12**), and 3 self-defined descriptors (Nos. **15**, **16**, **17**). Detailed information on the selected descriptors is given in Table 4 by using different models under different numbers.

Observing the relationship between descriptor types and algorithms reveals that different algorithms select different types of descriptors. The *MLRA* algorithm always chose descriptors from Codessa, PaDEL, and self-defined types under each number of descriptor from 7 to 3. The *RR* algorithm also chose descriptors from Codessa, PaDEL, and self-defined categories with the number of descriptors of 7, 6, and 5. The descriptors of PaDEL type become absent with the number of descriptors of 4 and 3. Moreover, different from *MLRA*, the proportion of PaDEL descriptors is obviously lower in the *RR* model. In contrast, *LASSO* and *EN* regression algorithms exclusively considered descriptors from Codessa and self-defined types, as shown in Table 4, excluding PaDEL descriptors.

The variation in selecting different types of descriptors via different ML models depends upon the regularization strengths applied to the loss function in these regression models. *LASSO* uses L1 regularization, whereas *RR* employs L2 regularization. The former adds a penalty term proportional to the summary of the absolute value of the coefficients for each descriptor, whereas the latter adds a penalty term proportional to the summary of the square of the coefficients for each descriptor, as described in Section 3 in detail. *EN* uses a hybrid regularization by combining both L1 and L2 to enhance model performance and handle correlated features effectively. The regularization parameter (α) tends to bring the coefficient values either close to zero (as in the *RR* model), or exactly equal to zero (as in the *LASSO* and *EN* models), and brings sparsity in the model by preventing overfitting. The descriptors that are considered unimportant in predicting the target variable are elucidated in this way. As the α in *RR* model brings coefficient values closer to zero but not exactly equal to zero, this is the potential reason for *RR* model to select all three types of descriptors under a large number of descriptors. On the other hand, the regularization applied in the *LASSO* and *EN* models brings the coefficient values equal to zero—thus, those unimportant descriptors (PaDEL descriptors in this case) are eliminated—and only selects descriptors from Codessa and self-defined types. Therefore, comparatively, the contributions by PaDEL descriptors are less important than that by Codessa and self-defined types.

As discussed in Section 2.4, considering the number of descriptors and performance of algorithm, the *RR* model containing 4 descriptors (Nos. **4**, **5**, **15**, **16)** is regarded as the optimum. Accordingly, to quantitatively detect the influence of descriptors on catalytic activity, the contribution for these 4 descriptors is calculated and listed in Table 5. The standardized values of descriptors and experimental catalytic activities of **Fe**/**Co** complexes for each descriptor are summarized in Table S12.

As can be seen in Table 5, it is clear that bite angle (*β*), a self-defined descriptor, shows the biggest contribution to catalytic activities with the value of 34.35%. As to the definition, bite angle (*β*) is calculated by the angle of coordination between the metal centre and the bonded nitrogen atoms (∠N1-M-N2), corresponding to the steric effect by the substituents. Usually, the bulky substituents lead to smaller bite angles, as seen in Table S5; *β* values decrease from 147.45 to 144.00 for complexes **Fe5** to **Fe8** as the R^2^ substituent varies from methyl, ethyl, iso-propyl to 2-methyl-ph. There is a small variation for complexes **Fe1** to **Fe4**, as the variation of substituents lies in the para-position within the N-aryl group, which is situated far from the metal centre. Therefore, a positive correlation with the activity indicates that the enlarged steric hindrance resulting from bulky substituents is conducive to reducing catalytic activity values, as it influences the accessibility of the ethylene monomer to the catalytic active sites.

RNCS relative negative charged SA (SAMNEG*RNCG) [31,32], a Codessa descriptor, positively correlates with the catalytic activity and shows a high contribution with a value of 29.20%**.** It represents the quantum-chemically calculated charge distribution in the molecules, reflecting a combination of the contributions of atomic negative charges to the total molecular solvent-accessible surface area. The value of this descriptor increases as the R^2^ substituent varies from methyl, ethyl, to iso-propyl for complexes **Fe5** to **Fe7**, owing to the increase in electron donating ability of substituents, as shown in Table S8. Correspondingly, an increase in activities is observed for complexes **Fe5** to **Fe7**.

Avg 1-electron react. index for a N atom [33], a quantum chemical Codessa descriptor, has negative coefficient values as shown in Table 5, and presents a relatively smaller contribution of 25.97% with activity. The descriptor is calculated as Equation (1):

(1) $$R_{A}=\sum_{i\epsilon A}\cdot \sum_{j\epsilon A}N_{i^{HOMO}}\cdot N_{j^{LUMO}}/(\varepsilon_{LUMO}-\varepsilon_{HOMO})$$

where ${\;N}_{j^{LUMO}}$ and $N_{i^{HOMO}}$ are the coefficients of the Lowest Unoccupied Molecular Orbitals (LUMO) and the Highest Occupied Molecular Orbitals (HOMO), and ${ɛ}_{LUMO}$ and ${ɛ}_{HOMO}$ are energies of these two molecular orbitals. This descriptor describes the relative reactivity of nitrogen atoms in complexes, and negatively correlates with catalytic activity.

Energy difference (Δ*E*), a self-defined type descriptor, is defined as the different optimized energies between spin states. It presents the contribution value of 10.48% to activity. The negative values for correlation suggest that a higher value of this descriptor is unfavorable for enhancement of catalytic activity, probably due to the high-energy barriers from one spin state to another one.

In summary, comparing different linear regression models for predicting catalytic activities, it is found that the *RR* model shows the optimal correlations and predicts the catalytic activities of 2-imino-1,10 phenanthrolyl **Fe**/**Co** complexes well. It reveals that different algorithms select different types of descriptors such as *MLRA* and *RR* models which consider descriptors from Codessa, PaDEL, and self-defined types, whereas the *LASSO* and *EN* models selected Codessa and self-defined descriptors, excluding PaDEL type. The dependence of descriptor type on the algorithm is mainly attributed to the regularization strengths employed in different linear algorithms. The interpretation of the *RR* model reveals that the most favorable factors for improved catalytic activity are less bulky substituents and higher negative charge distributions.

## 3. Computational Methods

### 3.1. Geometry Optimization

Geometries of all the complexes are optimized using the DFT method [34,35] under B3LYP hybrid exchange-correlation functional and 6-31G*(d) basis set using Gaussian Program Package [36]. A vibrational analysis is also performed at the same theory level to confirm that the optimized geometry represents a true minimum energy state with no imaginary frequencies. An electron distribution analysis is carried out by the use of natural bond orbital (NBO) analysis [37]. The structures of all the 16 complexes are optimized by considering their all-possible spin states. The geometries of iron (**Fe**) complexes are optimized at singlet, triplet, and quintet spin states, whereas for cobalt (**Co**) complexes, doublet and quartet spin states are considered. After successful optimizations, the structures of both the metal complexes at all possible spin states are compared with the experimental crystal structures to validate the optimization process.

### 3.2. Descriptor Calculation

To establish a quantitative relationship between the catalyst’s structure and its activity, molecular descriptors are calculated based on the optimized structures using Codessa and PaDEL programs. Seven self-defined descriptors are also calculated as reported earlier in our previous studies [16,17], and a brief description for each is given below:

The effective net charge is determined by the difference between the net charge of the metal atom and the difference in the net charges of halogen atoms bonded to it and is calculated with the equation given below:

(2) $$Q_{\mathrm{eff}}=Q_{\mathrm{CM}}-{\Delta Q}_{\mathrm{halogens}}$$

$Q_{\mathrm{CM}}$ represents net charge of the central metal atom and ${\Delta Q}_{\mathrm{halogens}}$ is the difference in net charges of two halogen atoms. The energy gap between the HOMO and LUMO levels, denoted as ${\Delta \varepsilon }_{1}$ and ${\Delta \varepsilon }_{2}$, respectively, represents the difference in energy between the complex’s LUMO/HOMO orbitals $\left(E_{\mathrm{LC}}/E_{\mathrm{HC}}\right)$ and the HOMO/LUMO orbitals of ethylene $\left(E_{\mathrm{HE}}/E_{\mathrm{LE}}\right)$. Equations (3) and (4) calculate it as follows:

(3) $${\Delta \varepsilon }_{1}=E_{\mathrm{LC}}-E_{\mathrm{HE}}$$

(4) $${\Delta \varepsilon }_{2}=E_{\mathrm{LE}}-E_{\mathrm{HC}}$$

The energy difference (ΔE) is described as the change in optimized energy between different spin states. The Hammett constant (F) serves as a parameter indicating the electronegativity of the substituents, and the values are obtained from the literature [38]. The open cone angle (*θ*) refers to the space surrounding the central metal in the complex, which allows for the accommodation of the incoming ethylene monomer, as proposed in our previous studies [15,16,17]. Additionally, the bite angle (*β*) represents the coordination angle between the metal and the bonded nitrogen atoms [39,40].

### 3.3. Feature Selection

After calculating the descriptors, it is required to reduce the number of features for modeling. So, as to the descriptors generated via Codessa software (version 2.7.2), the heuristic method (HM) is employed to select features based on two criteria such as by removing highly inter-correlated descriptors and by eliminating the least correlated descriptors with the target variable. Regarding the descriptors generated via PaDEL software (version 2.21), the PLS-VIP method is used to select descriptors based on the VIP value of 0.85. The dataset is divided into test–train sets in a 20:80 ratio by using the random selection method, which is complemented by the test–train split function in Python [41,42]. For better model predictions, different random states from 0 to 100 are tested and reasonable predictive scores for all the metrics are observed at random state 31. The GridSearch method with k-fold (k = 4) CV is used to optimize the parameter ‘number of components’. The robustness and accuracy of the PLS regression model is evaluated by computing correlation coefficients (*R*^2^), mean absolute error (*MAE*), root mean square error (*RMSE*), for both test and training sets, and cross-validation coefficients (*Q*^2^) using the shuffle split cross-validation method (n_splits = 4). Self-defined descriptors are selected based on the correlation (*R*^2^) values of each descriptor with the target variable.

### 3.4. Modeling

As to the limited number of observations in the dataset, linear machine learning algorithms are used for regression modeling. *MLRA* is utilized to analyze the relationships among various independent variables and dependent variables by using the following fitting equation:

(5) $$Act\cdot =\beta_{o}+\sum_{i=1}^{n}\beta_{i}x_{i}$$

where n is the number of descriptors, $\beta_{o}$ is value of the intercept, $\beta_{i}$ represents the coefficient value for each descriptor, and $x_{i}$ is the value of the $i^{th}$ descriptor.

Besides *MLRA*, *LASSO* and *RR* regression algorithms are also employed to address issues such as multicollinearity and overfitting. *LASSO* uses L1 regularization, while *RR* employs L2 regularization. The former adds a penalty term proportional to the summary of the absolute value of the coefficients for each descriptor, whereas the latter adds a penalty term proportional to the summary of the square of the coefficients for each descriptor. The loss functions for *LASSO* and *RR* are given, respectively, by following Equations (6) and (7), respectively:

(6) $$Minimize:\frac{1}{2n}\sum_{i=1}^{p}{\left(y_{i}-\beta_{0}-\sum_{j=1}^{n}\beta_{j}x_{ij}\right)}^{2}+\alpha \sum_{j=1}^{n}\left|\beta_{j}\right|$$

(7) $$Minimize:\frac{1}{2n}\sum_{i=1}^{p}{\left(y_{i}-\beta_{0}-\sum_{j=1}^{n}\beta_{j}x_{ij}\right)}^{2}+\alpha \sum_{j=1}^{n}{\beta_{j}}^{2}$$

Additionally, *EN* provides a versatile approach by incorporating both L1 and L2 regularizations to enhance model performance and handle correlated predictors effectively. Equation (8) is the loss function for *EN* regressions:

(8) $$Minimize:\frac{1}{2n}\sum_{i=1}^{p}{\left(y_{i}-\beta_{0}-\sum_{j=1}^{n}\beta_{j}x_{ij}\right)}^{2}+\alpha_{1}\sum_{j=1}^{n}\left|\beta_{j}\right|+\alpha_{2}\sum_{j=1}^{n}{\beta_{j}}^{2}$$

In the above equation, p is the number of observations, n is the number of descriptors, $y_{i}$ expresses the observed value for the $i^{th}$ observation, $\beta_{0}$ is the value of the intercept, $\beta_{j}$ is the coefficient value of the $j^{th}$ descriptor, $x_{ij}$ are the values of the $j^{th}$ descriptor for the $i^{th}$ observation, and α is the regularization parameter.

Based on the descriptors from three types, the final number of descriptors is selected by using the Recursive Feature Elimination (RFE) technique incorporated with linear models. RFE works on the principle of iterative refinement. It starts with the entire feature set, trains a model, evaluates feature importance, and systematically eliminates the least important features and thus by reducing the chance of overfitting it enhances the generalizability of linear models. The parameters for all the models are optimized via the GridSearch method with k-fold (k = 4) CV as shown in Table S13. α is explained as a hyper-parameter that controls the regularization strength to help prevent chances of overfitting. In *LASSO* and *RR* regressions, α represents L1 and L2 penalty terms, respectively, where higher values of α result in stronger regularizations. In *EN* regression model, α determines the trade-off between L1 and L2 regularization techniques; this means that values of α are in between 0 and 1 but not exactly equal to 0 or 1. The predictive performances for all the models are assessed by employing the shuffle–split cross-validation method available in Scikit-learn [41], where the dataset is randomly shuffled and divided into training and testing sets with the ratio 80:20 for each cross-validation iteration. In each iteration, a new set is employed as a test set and the remainder is training sets. This approach allows for a better generalization of the model’s performance.

## 4. Conclusions

In summary, the investigation into the catalytic activities of 2-imino-1,10-phenanthrolyl iron and cobalt metal complexes using machine learning (ML) methods has provided valuable insights into the influence of central metal and substituent effects. Incorporating descriptors from Codessa, PaDEL, and self-defined types, and employing four linear algorithms—*MLRA*, *LASSO*, *EN*, and *RR*—significant findings have been gained. Our study underscores the effectiveness of the *RR* model, which achieved optimal performance with 4 descriptors (*R*^2^ = 0.952, *Q*^2^ = 0.871). Additionally, the incorporation of descriptors from all the three different sources (Codessa, PaDEL, and self-defined types) in the *MLRA* and *RR* models underscores the robustness of the approach, enhancing the depth of the analysis. Through the interpretation of the *RR* model, the catalytic activity is correlated with the prevalence of less bulky substituents and negatively charged groups. This work reveals the preferences in descriptor selection for algorithmic preferences and provides insights into the underlying mechanisms of variation in catalytic activity. However, reliance on linear algorithms may introduce bias, and subjective descriptor selection limits generalizability. Despite these constraints, our study enriches catalysis and ML research, urging further exploration into nuanced modeling approaches.

## Figures and Tables

**Table 1:** Detailed information on each descriptor in the pool of selected descriptors; the sequence number of descriptors is highlighted in red, blue, and green for different types, respectively.

Sequence Number of Descriptors	Codessa Descriptors	Sequence Number of Descriptors	PaDEL Descriptors	Sequence Number of Descriptors	Self-Defined Descriptors
**1**	Min valency of a Cl atom	8	RDF40u	15	Energy difference (Δ*E*)
**2**	Highest normal mode vib frequency	9	RDF45v	16	Bite Angle (*β*)
**3**	Count of H-donors sites (quantum-chemical PC)	10	RDF50m	17	Open cone
					Angle (*θ*)
**4**	Avg 1-electron react. index for a N atom	11	SIC5		
**5**	RNCS relative negative charged SA (SAMNEG*RNCG) (quantum-chemical PC)	12	AATS7v		
**6**	Moment of inertia B	13	IC4		
**7**	Min (>0.1) bond order of a H atom	14	RDF45u		

**Table 2:** The prediction and validation performance of *MLRA* and *LASSO* models.

Number of Descriptors	MLRA				LASSO			
	*R* ^2^	*MAE ^a^*	*RMSE ^a^*	*Q* ^2^	*R* ^2^	*MAE ^a^*	*RMSE ^a^*	*Q* ^2^
**7**	0.973	2.544	2.841	0.817	0.949	3.114	3.926	0.755
**6**	0.945	3.574	4.073	0.758	0.949	3.114	3.926	0.755
**5**	0.911	4.261	5.200	0.610	0.942	3.503	4.206	0.747
**4**	0.911	4.287	5.203	0.673	0.921	4.038	4.886	0.746
**3**	0.831	5.700	7.172	0.547	0.917	4.080	5.031	0.743

*^a^* 10^4^ g·mol^−1^·h^−1^.

**Table 3:** The prediction and validation performance of *EN* and *RR* models.

Number of Descriptors	EN				RR			
	*R* ^2^	*MAE ^a^*	*RMSE ^a^*	*Q* ^2^	*R* ^2^	*MAE ^a^*	*RMSE ^a^*	*Q* ^2^
**7**	0.994	1.044	1.316	0.944	0.996	0.844	1.165	0.960
**6**	0.966	2.723	3.216	0.840	0.995	0.952	1.253	0.954
**5**	0.950	3.399	3.882	0.774	0.963	2.903	3.348	0.899
**4**	0.949	3.515	3.932	0.886	0.952	3.597	3.817	0.871
**3**	0.942	3.797	4.191	0.879	0.942	3.789	4.185	0.882

*^a^* 10^4^ g·mol^−1^·h^−1^.

**Table 4:** Information on descriptors selected by different models under different numbers; the sequence numbers highlighted in red, blue, and green stand for Codessa, PaDEL, and self-defined types, respectively.

Number of Descriptors	MLRA	LASSO	EN	RR
**7**	**3**, **8**, **11**, **12**, **15**, **16**, **17**	**1**, **3**, **4**, **5**, **6**, **16**, **17**	**1**, **3**, **4**, **5**, **6**, **16**, **17**	**3**, **4**, **5**, **9**, **11**, **15**, **16**
**6**	**3**, **8**, **11**, **12**, **16**, **17**	**1**, **3**, **4**, **5**, **6**, **16**	**1**, **4**, **5**, **6**, **16**, **17**	**3**, **4**, **5**, **11**, **15**, **16**
**5**	**3**, **8**, **11**, **12**, **16**	**1**, **3**, **4**, **5**, **16**	**1**, **4**, **5**, **16**, **17**	**4**, **5**, **11**, **15**, **16**
**4**	**3**, **8**, **11**, **16**	**1**, **3**, **5**, **16**	**1**, **4**, **5**, **16**	**4**, **5**, **15**, **16**
**3**	**8**, **11**, **16**	**1**, **3**, **16**	**4**, **5**, **16**	**4**, **5**, **16**

**Table 5:** Regression coefficients and percent contribution of important descriptors for the optimal model.

Sequence Number of Descriptors	Molecular Descriptors	Type	Coefficients	Contribution%
**16**	Bite angle (*β)*	Self-defined	0.855	34.35
**5**	RNCS relative negative charged SA (SAMNEG*RNCG)	Codessa	0.521	29.20
**4**	Avg 1-electron react. index for a N atom	Codessa	−0.424	25.97
**15**	Energy difference (Δ*E*)	Self-defined	−0.206	10.48

## Data Availability

The raw data supporting the conclusions of this article will be made available by the authors, without undue reservation.

## Supplementary Materials

The following are available online at https://www.mdpi.com/article/10.3390/molecules29102313/s1, Tables S1–S4: Comparisons of bond lengths and bond angles between calculated geometry and experimental values for complexes **Fe7**, **Co3**, **Co7**, and **Co8** along with standard deviation (δ) at various spin states, respectively; Table S5: The values of Hammett constant (*F*), effective net charge (Q_eff_), open cone angle (*θ*), bite angle (*β*), energy difference (Δ*E*), HOMO-LUMO energy gap (${\Delta \varepsilon }_{1}$, ${\Delta \varepsilon }_{2}$), for **Fe**/**Co** complexes; Table S6: Pearson correlation coefficient values of self-defined descriptors with catalytic activity; Table S7: The highest correlations values (*R*^2^) for different numbers of descriptors; Table S8: The detailed information of the 7 descriptors calculated by Codessa; Table S9: The values of *R*^2^*, MAE^a^*, *RMSE^a^,* and *Q*^2^ for PLS model at different number of descriptors; Table S10: The detailed information of the 7 descriptors by PaDEL; Table S11: Predicted catalytic activities by different linear machine learning models; Table S12: Standardized values of descriptors and experimental catalytic activities of **Fe**/**Co** complexes along with the percentage contribution of each descriptor; Table S13: Optimizing parameters grid by GridSearchCV method for the four linear algorithms; Figure S1: The triangular matrix of the correlations among 17 selected descriptors and activity.
