# Supplementary material for: Catalytic Activity of 2-Imino-1,10-phenthrolyl Fe/Co Complexes via Linear Machine Learning
Source: Molecules. 2024 May 15;29(10):2313. doi: 10.3390/molecules29102313 (PMC11124342; doi:10.3390/molecules29102313)
Supplement: Supplementary file 1 [file molecules-29-02313-s001.zip › molecules-2961195-supplementary.pdf]

Catalytic Activity of 2-imino-1,10-phenthrolyl Fe/Co Complexes by Linear Machine Learning

Zubair Sadiq,[1,2] Wenhong Yang,[3,*], Md Mostakim Meraz[1,2], Weisheng Yang,[3] and Wen-Hua Sun[1,2,*]

[1]Key laboratory of Engineering Plastics, Beijing National Laboratory for Molecular Science, Institute of Chemistry, Chinese Academy of Sciences, Beijing 100190, China

[2]University of Chinese Academy of Sciences, Beijing 100049, China

[3]PetroChina Petrochemical Research Institute, Beijing 102206, China

Correspondence to: Wenhong Yang (E-mail: whyang@iccas.ac.cn); Wen-Hua Sun (E-mail: whsun@iccas.ac.cn)

Table S1. Comparisons of bond lengths and bond angles between calculated geometry and experimental values for complex **Fe7** along with standard deviation (δ) at various spin states.

**Bond Lengths (Å)**				
**Fe7**	**Experimental**	**Singlet**	**Triplet**	**Quintet**
Fe1-N1	2.26	2.02	2.09	2.29
Fe1-N2	2.09	1.82	1.85	2.04
Fe1-N3	2.28	2.02	1.99	2.18
Fe1-Cl1	2.28	2.24	2.25	2.26
Fe1-Cl2	2.28	2.26	2.30	2.30
N3-C13	1.29	1.31	1.33	1.31
N3-C15	1.45	1.45	1.44	1.44
**δ**		6.35	6.32	2.16
**Bond Angles (°)**				
N1-Fe1-N2	75.16	82.59	80.22	74.30
N1-Fe1-N3	147.41	162.07	152.17	145.20
N2-Fe1-N3	72.89	80.08	79.10	74.70
N1-Fe1-Cl2	97.20	90.90	93.10	92.30
N2-Fe1-Cl2	116.43	139.02	157.18	143.80
N3-Fe1-Cl2	102.52	99.18	98.79	103.70
N1-Fe1-Cl1	93.02	88.70	94.75	95.80
N2-Fe1-Cl1	121.90	91.75	95.81	96.80
N3-Fe1-Cl1	98.33	96.33	105.67	103.10
Cl2-Fe1-Cl1	121.48	128.65	106.51	118.20
**δ**		12.37	14.94	10.81

Table S2. Comparisons of bond lengths and bond angles between calculated geometry and experimental values for complex **Co3** along with standard deviation (δ) at various spin states.

**Bond Lengths (Å)**			
**Co3**	**Experimental**	**Doublet**	**Quartet**
Co1-N1	2.17	2.00	2.22
Co1-N2	2.06	1.84	2.04
Co1-N3	2.24	1.99	2.25
Co1-Cl1	2.30	2.40	2.31
Co1-Cl2	2.24	2.23	2.28
N3-C13	1.29	1.31	1.29
N3-C15	1.45	1.45	1.44
**δ**		6.91	1.06
**Bond Angles (°)**			
N1-Co1-N2	76.68	82.28	75.78
N1-Co1-N3	145.33	160.39	144.16
N2-Co1-N3	72.86	79.91	73.73
N1-Co1-Cl2	100.63	94.09	94.05
N2-Co1-Cl2	152.03	157.46	145.44
N3-Co1-Cl2	98.57	99.19	100.44

N1-Co1-Cl1	94.20	88.33	95.71
N2-Co1-Cl1	93.36	90.54	92.22
N3-Co1-Cl1	103.64	99.94	103.79
Cl2-Co1-Cl1	114.60	111.65	121.90
**δ**		6.18	3.53

Table S3. Comparisons of bond lengths and bond angles between calculated geometry and experimental values for complex **Co7** along with standard deviation (δ) at various spin states.

**Bond Lengths (Å)**			
**Co7**	**Experimental**	**Doublet**	**Quartet**
Co1-N1	2.23	1.99	2.22
Co1-N2	2.03	1.84	2.05
Co1-N3	2.27	1.98	2.25
Co1-Cl1	2.24	2.23	2.28
Co1-Cl2	2.26	2.38	2.30
N3-C13	1.29	1.31	1.29
N3-C15	1.44	1.45	1.44
**δ**		7.19	0.96
**Bond Angles (°)**			
N1-Co1-N2	76.44	82.29	75.77
N1-Co1-N3	150.56	160.74	144.28
N2-Co1-N3	74.52	79.97	73.65
N1-Co1-Cl2	95.35	89.35	96.95
N2-Co1-Cl2	115.93	90.62	92.36
N3-Co1-Cl2	101.50	98.25	102.29
N1-Co1-Cl1	93.16	93.81	93.26
N2-Co1-Cl1	122.39	156.25	143.90
N3-Co1-Cl1	98.27	99.40	100.64
Cl2-Co1-Cl1	121.45	112.81	123.35
**δ**		12.85	9.15

Table S4. Comparisons of bond lengths and bond angles between calculated geometry and experimental values for complex **Co8** along with standard deviation (δ) at various spin states.

**Bond Lengths (Å)**			
**Co8**	**Experimental**	**Doublet**	**Quartet**
Co1-N1	2.16	2.00	2.22
Co1-N2	2.06	1.84	2.04
Co1-N3	2.25	1.98	2.25
Co1-Cl1	2.30	2.40	2.31
Co1-Cl2	2.24	2.23	2.28
N3-C13	1.28	1.31	1.29
N3-C15	1.43	1.45	1.43
**δ**		6.57	1.07

Bond Angles (°)			
N1-Co1-N2	76.74	82.29	75.82
N1-Co1-N3	144.76	160.33	144.02
N2-Co1-N3	72.64	79.90	73.64
N1-Co1-Cl2	101.40	94.18	94.25
N2-Co1-Cl2	153.10	157.92	145.84
N3-Co1-Cl2	98.13	99.17	100.38
N1-Co1-Cl1	93.46	88.34	95.73
N2-Co1-Cl1	93.46	90.34	92.10
N3-Co1-Cl1	105.27	100.00	103.91
Cl2-Co1-Cl1	113.43	111.40	121.65
δ		6.58	3.98

Table S5. The values of Hammett constant (F), effective net charge (Q_{eff}), open cone angle (θ), bite angle (β), energy difference (ΔE), HOMO-LUMO energy gap ($\Delta\varepsilon_1$, $\Delta\varepsilon_2$), for **Fe**/**Co** complexes.

Complexes	Descriptors						
	F	Q_{eff}	θ (°)	β (°)	ΔE [kcal/mol]	$\Delta\varepsilon_1$ [kcal/mol]	$\Delta\varepsilon_2$ [kcal/mol]
**Fe1**	0.02	0.61	66.79	143.99	13.06	106.31	117.93
**Fe2**	0.01	0.61	66.74	143.97	13.06	106.26	117.97
**Fe3**	0.05	0.62	66.88	143.93	13.06	106.29	117.97
**Fe4**	0.43	0.62	67.05	143.99	13.02	103.75	120.51
**Fe5**	0.02	0.63	74.27	147.45	14.00	105.91	118.84
**Fe6**	0.01	0.63	74.58	147.25	14.11	105.87	118.88
**Fe7**	0.05	0.62	79.52	145.20	13.28	106.04	118.69
**Fe8**	0.02	0.62	66.23	144.00	13.09	106.06	118.17
**Co1**	0.02	0.50	65.66	144.07	12.01	102.03	140.71
**Co2**	0.01	0.49	66.25	144.24	12.02	101.99	140.75
**Co3**	0.05	0.50	65.67	144.18	12.01	101.98	140.76
**Co4**	0.43	0.51	66.74	144.37	12.05	99.44	143.27
**Co5**	0.02	0.52	79.06	144.63	12.83	102.00	139.02
**Co6**	0.01	0.51	81.77	144.49	12.62	102.25	138.77
**Co7**	0.05	0.51	77.31	144.28	12.42	102.27	139.15
**Co8**	0.02	0.50	65.47	144.03	12.03	101.78	140.98

Table S6. Pearson correlation coefficient values of self-defined descriptors with catalytic activity.

Names of descriptors	Correlation with activity (R^2)
β	0.819
ΔE	0.278
θ	0.253
Q_{eff}	0.043
F	0.034
$\Delta\varepsilon_1$	0.033

$\Delta\varepsilon_2$	0.023

Table S7. The highest correlations values (R^2) for different numbers of descriptors.

Descriptor Combinations	R^2
7	0.991
6	0.974
5	0.961
4	0.944
3	0.930

Table S8. The detailed information of the 7 descriptors calculated by Codessa.

Complex	Min valency of a Cl atom	Highest normal mode vib frequency	count of H-donors sites [Quantum-Chemical PC]	Avg 1-electron react. index for a N atom	RNCS Relative negative charged SA (SAMNEG*RNCG) [Quantum-Chemical PC]	Moment of inertia B	Min (>0.1) bond order of a H atom
**Fe1**	0.637	3233.9	8	1.9E-04	0.259	0.002	0.851
**Fe2**	0.637	3233.9	10	1.8E-04	0.249	0.002	0.851
**Fe3**	0.637	3233.8	12	1.9E-04	0.240	0.002	0.851
**Fe4**	0.640	3234.1	5	2.5E-04	0.275	0.002	0.850
**Fe5**	0.659	3231.3	8	-2.5E-04	0.217	0.001	0.854
**Fe6**	0.659	3231.0	10	2.1E-04	0.292	0.001	0.854
**Fe7**	0.654	3232.9	12	1.5E-04	0.362	0.001	0.868
**Fe8**	0.637	3233.8	6	1.2E-04	0.185	0.001	0.852
**Co1**	0.647	3239.0	8	-1.1E-04	0.131	0.002	0.854
**Co2**	0.647	3239.0	10	-1.2E-04	0.126	0.002	0.854
**Co3**	0.647	3239.0	12	-1.2E-04	0.203	0.001	0.854
**Co4**	0.650	3239.4	5	-2.0E-04	0.186	0.002	0.852
**Co5**	0.646	3237.1	8	3.7E-04	0.309	0.001	0.861
**Co6**	0.646	3237.1	10	1.3E-04	0.254	0.001	0.865
**Co7**	0.646	3236.7	12	1.2E-04	0.326	0.001	0.864
**Co8**	0.647	3238.8	6	-6.8E-05	0.150	0.001	0.854

Table S9. The values of R^2, *MAE* [a], *RMSE* [a], and Q^2 for PLS model at different number of descriptors.

No. of descriptors	No. of components	R^2 -Train	R^2 -Test	*MAE* [a] -Train	*MAE* [a] -Test	*RMSE* [a] -Train	*RMSE* [a] -Test	Q^2 (n_splits=4)
381	4	0.9396	0.6988	3.15	11.49	3.74	12.81	0.5945
326	3	0.9234	0.6562	3.56	11.78	4.12	13.67	0.6310
311	3	0.9213	0.6977	3.59	11.26	4.24	12.81	0.6527
289	3	0.9261	0.7188	3.40	11.10	4.12	12.37	0.6682
270	3	0.9264	0.7385	3.55	10.87	4.00	11.92	0.6828

199	2	0.8240	0.6005	4.64	12.50	6.24	14.73	0.5699
189	2	0.7792	0.5283	4.77	13.49	7.00	16.00	0.5488
182	2	0.7700	0.4946	4.90	14.02	7.14	16.58	0.5659
180	2	0.7519	0.4635	5.07	14.48	7.48	17.09	0.5518
7	1	0.5109	0.2587	8.14	18.41	10.44	20.07	0.5935

[a] 10^4 g·mol^{-1}·h^{-1}

Table S10. The detailed information of the 7 descriptors by PaDEL.

Complex	RDF40u	RDF45v	RDF50m	SIC5	AATS7v	IC4	RDF45u
**Fe1**	62.991	41.782	80.879	0.754	233.387	4.835	69.578
**Fe2**	64.034	42.894	81.132	0.761	233.902	4.914	71.793
**Fe3**	66.339	45.544	81.515	0.753	234.373	4.899	82.721
**Fe4**	63.740	42.216	78.360	0.752	243.040	4.825	68.961
**Fe5**	55.675	35.851	66.053	0.792	214.981	5.031	59.943
**Fe6**	57.284	36.266	69.120	0.797	213.119	5.104	60.015
**Fe7**	57.705	37.070	73.481	0.788	211.563	5.082	60.435
**Fe8**	72.878	51.253	96.311	0.704	241.500	4.655	85.496
**Co1**	57.470	37.613	68.926	0.754	212.419	4.835	67.510
**Co2**	58.189	38.628	69.650	0.761	212.792	4.914	68.703
**Co3**	60.890	40.675	69.931	0.753	213.126	4.899	76.521
**Co4**	57.864	38.137	67.811	0.752	221.718	4.825	63.976
**Co5**	52.121	36.706	60.270	0.792	203.946	5.031	59.677
**Co6**	54.373	37.266	64.946	0.797	204.221	5.104	61.855
**Co7**	53.973	38.264	69.597	0.788	204.459	5.082	62.304
**Co8**	69.714	45.744	88.635	0.704	224.383	4.655	78.866

Table S11. Predicted catalytic activities by different linear machine learning models.

Catalytic Activities [a]					
**Complex**	**Expt.**	*MLRA*	*LASSO*	*EN*	*RR*
**Fe1**	5.9	7.2	6.1	8.2	8.6
**Fe2**	7.3	8.6	8.4	7.5	7.5
**Fe3**	8.8	12.9	9.8	5.4	5.2
**Fe4**	2.5	1.0	6.0	9.0	8.2
**Fe5**	61.0	60.5	55.1	63.4	63.9
**Fe6**	55.0	58.7	56.2	51.2	51.4
**Fe7**	42.0	33.1	38.2	41.7	39.7
**Fe8**	5.5	6.7	2.9	2.2	1.4
**Co1**	7.1	16.1	15.0	11.3	10.7
**Co2**	15.0	20.9	18.8	12.6	12.5
**Co3**	25.8	24.4	21.1	20.9	22.1

**Co4**	20.5	13.3	17.5	25.4	25.5
**Co5**	13.7	19.1	20.8	16.8	17.4
**Co6**	14.8	17.3	21.2	19.3	19.8
**Co7**	32.6	23.1	22.3	26.0	27.8
**Co8**	14.8	11.5	12.8	11.4	10.7

[a] 10^4 g·mol^{-1}·h^{-1}

Table S12. Standardized values of descriptors and experimental catalytic activities of **Fe**/**Co** complexes along with the percentage contribution of each descriptor.

**Complex systems**	**Complex no.**	**Bite angle (*β*)**	**RNCS Relative negative charged SA (SAMNEG*RNCG)**	**Avg 1-electron react. index for a N atom**	**Energy difference (Δ*E*)**	**Activity** [a]
**Fe-Cl**	**Fe1**	-0.579	0.343	0.667	0.393	-0.826
	**Fe2**	-0.591	0.202	0.606	0.394	-0.748
	**Fe3**	-0.628	0.068	0.667	0.397	-0.665
	**Fe4**	-0.575	0.572	1.031	0.340	-1.015
	**Fe5**	2.534	-0.261	-1.732	1.777	2.235
	**Fe6**	2.362	0.819	0.816	1.941	1.902
	**Fe7**	0.517	1.825	0.451	0.716	1.179
	**Fe8**	-0.570	-0.728	0.291	0.445	-0.848
**Co-Cl**	**Co1**	-0.505	-1.500	-0.981	-1.146	-0.759
	**Co2**	-0.354	-1.572	-0.101	-1.142	-0.320
	**Co3**	-0.400	-0.470	-1.003	-1.152	0.280
	**Co4**	-0.234	-0.713	-1.439	-1.097	-0.015
	**Co5**	-0.004	1.063	1.706	0.062	-0.393
	**Co6**	-0.123	0.275	0.340	-0.257	-0.332
	**Co7**	-0.310	1.314	0.324	-0.541	0.657
	**Co8**	-0.540	-1.236	-0.735	-1.128	-0.332
**Contribution percentage (%)**		34.35	29.20	25.97	10.48	

[a] 10^4 g·mol^{-1}·h^{-1}

Table S13. Optimizing parameters grid by GridSearchCV method for the four linear algorithms.

**ML Models**	**Parameters Grid**	**Optimal Parameters**
RR	α: [0.00001, 0.0001, 0.001, 0.01, 0.1, 1.0, 10.0, 100.0, 1000.0]	α=0.01
EN	α: [0.01, 0.1, 0.2, 0.4, 0.6, 0.8, 0.9, 1.0, 1.1, 1.2, 1.3, 2, 10, 100], l1_ratio: [0.01, 0.1, 0.2, 0.4, 0.6, 0.8, 0.9, 1.0]	α=0.01, l1_ratio=0.01
LASSO	α: [0.001, 0.01, 1.0, 1.10, 1.20, 1.30, 1.40, 1.50, 1.80, 2, 10.0, 100.0]	α=1.0

MLRA	non-parametric

	No.1	No.2	No.3	No.4	No.5	No.6	No.7	No.8	No.9	No.10	No.11	No.12	No.13	No.14	No.15	No.16	No.17	Act.
No.1	1.00																	
No.2	0.02	1.00																
No.3	0.01	0.02	1.00															
No.4	0.17	0.16	0.02	1.00														
No.5	0.01	0.29	0.15	0.49	1.00													
No.6	0.21	0.06	0.01	0.01	0.10	1.00												
No.7	0.13	0.01	0.23	0.05	0.34	0.34	1.00											
No.8	0.27	0.09	0.41	0.06	0.44	0.08	0.44	1.00										
No.9	0.52	0.01	0.07	0.02	0.09	0.01	0.24	0.62	1.00									
No.10	0.28	0.08	0.25	0.05	0.39	0.03	0.33	0.95	0.74	1.00								
No.11	0.44	0.01	0.01	0.01	0.23	0.04	0.31	0.61	0.87	0.74	1.00							
No.12	0.42	0.11	0.21	0.03	0.03	0.15	0.53	0.49	0.62	0.45	0.46	1.00						
No.13	0.34	0.01	0.11	0.01	0.14	0.01	0.34	0.68	0.90	0.79	0.82	0.71	1.00					
No.14	0.35	0.04	0.08	0.02	0.05	0.01	0.19	0.56	0.88	0.68	0.68	0.66	0.93	1.00				
No.15	0.08	0.94	0.01	0.15	0.29	0.11	0.01	0.16	0.01	0.17	0.07	0.03	0.01	0.01	1.00			
No.16	0.70	0.34	0.01	0.02	0.07	0.22	0.02	0.31	0.30	0.33	0.32	0.13	0.17	0.17	0.52	1.00		
No.17	0.21	0.07	0.14	0.13	0.51	0.41	0.75	0.69	0.36	0.63	0.52	0.45	0.48	0.32	0.14	0.21	1.00	
Act.	0.78	0.19	0.11	0.07	0.11	0.27	0.13	0.38	0.33	0.33	0.32	0.26	0.22	0.19	0.28	0.82	0.25	1.00

Figure S1. The triangular matrix of the correlations among 17 selected descriptors and activity.
